# Coconut oil supplementation reduces blood pressure and oxidative stress in spontaneously hypertensive rats

**DOI:** 10.1186/1753-6561-8-S4-P68

**Published:** 2014-10-01

**Authors:** Naiane Ferraz Bandeira Alves, Naiane Alves, Suênia Porpino, Matheus Monteiro, Thyago Queiroz, Karen Montenegro, Valdir Braga

**Affiliations:** 1 Universidade Federal Da Paraíba, João Pessoa, PB, Brazil

## Background

Oxidative stress has been implicated in the pathogenesis of hypertension and antioxidant compounds have been used in the prevention and treatment of this disease. In this context, coconut oil (CO) was found to have antioxidant property due to its high polyphenol content. In this study, we investigated the effects caused by chronic treatment with CO on mean arterial pressure (MAP), heart rate (HR), and lipid peroxidation in spontaneously hypertensive rats (SHR).

## Methods

SHR and their controls (Wistar Kyoto, WKY) were orally treated with coconut oil (2 ml/day) or saline for thirty days. Cardiovascular parameters were evaluated 24 hours after the end of the thirtieth day. Serum oxidative stress was measured by tiobarbituric acid reactive species assay (TBARS).

## Results and conclusions

The administration of CO was able to reduced the MAP of SHR compared to SHR treated with saline (146 ± 11 n = 5 *vs*. 174 ± 6 n = 8 mmHg, respectively, p < 0.05). On the other hand, treatment with CO had no effect on MAP of WKY compared with WKY treated with saline (136 ± 3 n = 6 *vs*. 113 ± 1 mmHg n = 8, respectively, p < 0.05). In addition, CO supplementation reduced lipid peroxidation in the SHR group compared to SHR + saline (13.7 ± 2 *vs*. 27.5 ± 5 nmol/g n = 7, respectively, p < 0.05) and also reduced in WKY compared with WKY treated with saline (11.5 ± 1 *vs*. 21.9 ± 2 nmol/g n = 7 respectively, p < 0.05). We concluded that treatment with coconut oil (2 ml/day) for thirty days reduces the blood pressure and oxidative stress in hypertensive rats.
